# Synthesis of Self-Assembled Nanostructured Cisplatin Using the RESS Process

**DOI:** 10.3390/pharmaceutics16111471

**Published:** 2024-11-18

**Authors:** Sudhir Kumar Sharma, Loganathan Palanikumar, Renu Pasricha, Thirumurugan Prakasam, Mazin Magzoub, Ramesh Jagannathan

**Affiliations:** 1 Engineering Division, New York University Abu Dhabi, Abu Dhabi P.O. Box 129188, United Arab Emirates; sks16@nyu.edu; 2 Biology Program, Division of Science, New York University Abu Dhabi, Abu Dhabi P.O. Box 129188, United Arab Emirates; pl105@nyu.edu (L.P.); mm6432@nyu.edu (M.M.); 3 Retired from Core Technology Platform, New York University Abu Dhabi, Abu Dhabi P.O. Box 129188, United Arab Emirates; renu1505@gmail.com; 4 Science Division, New York University Abu Dhabi, Abu Dhabi P.O. Box 129188, United Arab Emirates; tp42@nyu.edu

**Keywords:** supercritical CO_2_ processing, RESS technique, liquid in situ transmission electron microscopy, cell viability, apoptosis

## Abstract

**Background/Objectives:** The primary goal of our research is to develop a process to prepare an aqueous dispersion of Cisplatin, an important anticancer drug, with increased solubility and storage stability. **Method:** In this context, we report the use of a customized RESS process for the synthesis of a novel, amber-colored and viscous aqueous cisplatin solution, an important anticancer drug, which we have denoted as “liquid” cisplatin. **Results:** Using specialized liquid cell in situ transmission electron microscopy (Liquid in situ TEM) and Raman spectroscopy, we demonstrated that “liquid” cisplatin comprises a bi-modal distribution of a highly solvated network of stable cisplatin nanoclusters in water and exhibited 27 times greater water solubility than standard cisplatin. More importantly, “liquid” cisplatin was stable at ambient conditions for over two years. Extensive analytical characterization of “liquid” cisplatin confirmed that it retained the original chemical identity of cisplatin. Cell viability and apoptosis studies on human lung adenocarcinoma A549 cells provided compelling evidence that “liquid” cisplatin demonstrated a more sustained anticancer effect compared to standard cisplatin. **Conclusions:** Aqueous cisplatin solubility was increased by 27X in the “liquid” cisplatin medium which retained its bio efficacy over a 2-year period. Our experimental results suggest the possibility of developing non-invasive and highly effective novel cisplatin drug-delivery platforms.

## 1. Introduction

According to the World Health Organization (WHO) report on cancer (i.e., malignant tumors and neoplasms), it is a leading cause of death globally, and results in over 10 million deaths annually. Lung, colon/rectum, liver, stomach and breast cancers lead the list. In terms of new occurrences, breast, lung, colon/rectum, prostate, skin and stomach cancers are the most dominant ones, in that order. Annually, over 40,000 children develop cancer. The cancer burden is expected to increase by 60% over the next two decades [1]. Clearly, these are devastating statistics and there is general consensus among the medical community that early detection, appropriate treatment and proper aftercare of patients who develop cancer are key to significantly reducing the cancer burden.

For example, a remarkable 65% decrease in the occurrence of cervical cancer was observed among women in their early 20s between 2012 and 2019. They were the first group who received the human papillomavirus vaccine. Despite the pandemic, and in contrast with other leading causes of death, the cancer death rate continued to decline from 2019 to 2020 (by 1.5%), contributing to a 33% overall reduction since 1991 and an estimated 3.8 million deaths were averted [2]. In general, the number of cancer survivors continues to increase in the United States due to the growth and aging of the population as well as advances in early detection and treatment. More specifically, these data are encouraging in terms of advances in cancer treatment, which are particularly evident in the rapid declines in occurrences and mortality (approximately 2% annually during 2016 through 2020) for leukemia, melanoma and kidney cancer, despite stable/increasing incidence, and accelerated declines for lung cancer. More than 18 million Americans (8.3 million males and 9.7 million females) with a history of cancer were alive on 1 January 2022. More than one-half (53%) of survivors were diagnosed within the past 10 years, and two-thirds (67%) were aged 65 years or older [3]. 

In cancer treatment, it is important to recognize a major advance that was due to the accidental discovery of the inhibition of *Escherichia coli* cell division in electrolysis experiments using platinum electrodes by Petsko [4]. Cis-dichloroammineplatinum (II), namely, cisplatin, was identified as the electrolysis product that was responsible for cell inhibition and became the first platinum compound that was FDA approved for cancer treatment in 1978 [5,6,7,8]. Since then, cisplatin has rapidly become the most commonly used intravenously administered chemotherapy drug for a broad range of cancer types, such as, leukemia, lymphomas, breast, testicular, ovarian, head and neck, cervical and sarcomas. The anticancer activity of cisplatin is due to its ability to bind with the DNA and disrupt its repair mechanisms leading to DNA damage, and subsequent cancer cell apoptosis [9]. Moreover, it restricts the protein synthesis and cell proliferation. In spite of its massive success, cisplatin has severe side effects and its clinical utility is limited by toxicological and tumor-resistance considerations [10]. Treatment with cisplatin also causes severe side effects like kidney damage, allergic effects, compromised immunity to infections, gastrointestinal disorders, hemorrhage and hearing loss [11]. 

Most of the therapies used for malignancies lack target specificity and thus require a high dose administration, which leads to extended tissue toxicity. Cisplatin is no exception and results in nephrotoxicity, neurotoxicity and ototoxicity with cytotoxic effects [12]. The activity and side effects of cisplatin treatments depend on pharmacological parameters like the dosage type (single or cumulative) and administration (schedule and means) and systemic and individual factors such as skin pigmentation, age, blood pH and diet [13]. Cisplatin exhibited variable cytotoxic response due to specific handling procedures and storage conditions which affected its chemical stability [4,14]. In laboratory scale and clinical setups, the stock solution of cisplatin is usually prepared in organic solvents like dimethyl formamide (DMF) or phosphate buffered saline (PBS) (up to 100 mM concentration). Although cisplatin is highly soluble in dimethyl sulfoxide (DMSO), the solvent’s sulfur group reduces the biological activity of cisplatin or deactivates it [15]. In addition to its poor water solubility (1 mg/mL), cisplatin is highly unstable as an aqueous solution and is usually formulated in a high-concentration saline solution for clinical use (154 mM) to prevent drug inactivation prior to intravenous administration [16,17,18]. Saline solutions of cisplatin are typically stable for up to 30 days at room temperature, in light shielded containers.

Given the two significant constraints of poor water solubility and chemical instability of cisplatin, we focused our efforts on enhancing cisplatin water solubility through the well-known phenomenon of particle size reduction (i.e., micronization) and used the customized rapid expansion of supercritical solutions (RESS) process platform developed in our laboratory [19]. Among various conventional micronization strategies, supercritical fluid-based technologies are found to be very promising as they are scalable, non-toxic, solvent-free and environmentally compatible [20]. In this manuscript, we report on our successful effort to increase the aqueous solubility of cisplatin by **27×** through the formation of a network of stable cisplatin nanoclusters in water. The resulting water solution was an amber-colored viscous liquid, herein referred to as “liquid” cisplatin, that was stable at room temperature for over a year while exhibiting a more sustained anticancer effect compared to conventional cisplatin formulations. This has the potential to open up novel and non-invasive drug-delivery applications of this very important cancer-treatment drug.

## 2. Materials and Methods

### 2.1. Synthesis of “Liquid” Cisplatin via Rapid Expansion of Supercritical Solvents (RESS) Process

**Figure 1:**
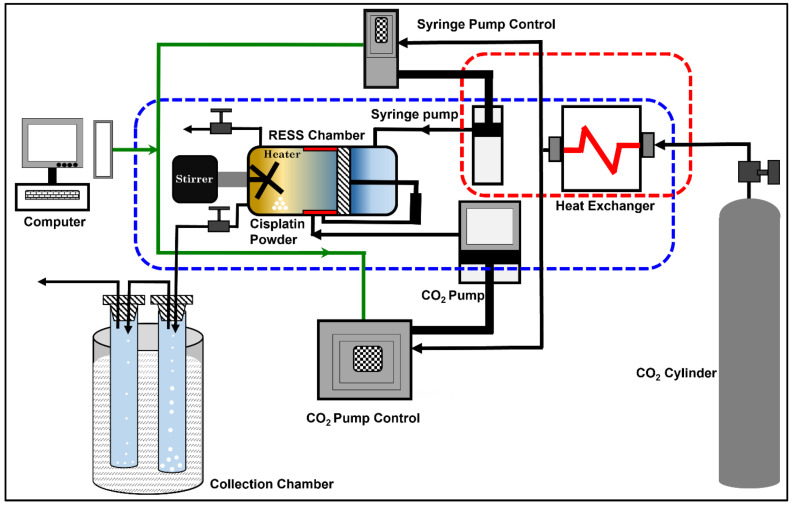
A schematic of RESS technique. A custom designed sc-CO_2_-based rapid expansion of supercritical solutions (RESS technique) was used to synthesize nanostructured “liquid” cisplatin.

The conventional cisplatin powder was procured from Sigma-Aldrich (PO Box 14508, St. Louis, MO 63178, USA). In our RESS experiments, the process chamber is first loaded with 100 mg of standard cisplatin powder and sealed tightly. Liquid carbon dioxide (purity 99.9%) was injected into the process chamber using a syringe pump. Total mass of liquid carbon dioxide used for each experiment was 212 g. The operating pressure of the process chamber was maintained constant to 300 bar by a back pressure-controlled CO_2_ pump. The high pressure of 300 bar was required to solubilize cisplatin in sc-CO_2_ in a reasonable length of time. In built cartridge heaters were used to maintain a chamber temperature of 40 °C. The above operating pressure and temperature were selected so that the liquid carbon dioxide will convert into supercritical state i.e., supercritical CO_2_ (sc-CO_2_). The mixer with rotation speed of 120 rpm was used to mix the cisplatin powder with sc-CO_2_ so that it is completely dissolved in the supercritical medium. This mixing process was allowed to continue for 6 h and the system was then allowed to equilibrate for 30 min, after turning off the mixer. The outlet of the process chamber was connected with stainless steel capillary tube (125 μm diameter) with a manually operated needle valve. Afterword’s, the process chamber was depressurized by slightly opening the needle valve while maintaining constant pressure and temperature within the process chamber. The outlet of the capillary tube was directed into a cold trap (45 mm OD × 250 mm overall length; 16 mm OD side and inner tubes, 40/50 joint) cooled below the freezing point of solid carbon dioxide (T ≤ −78 °C, coolant medium: liquid nitrogen). The rapid gasification and reduction in the density of CO_2_ resulted in a turbulent, supersonic jet stream and significant Joules–Thomson cooling. The high supersaturation induced rapid precipitation resulted in cisplatin nanoclusters. [21] The cold trap was then removed from the liquid N_2_ bath and brought to room temperature when a semi-transparent viscous liquid, hereafter referred to as “liquid” cisplatin was observed. The viscous “liquid” cisplatin was further concentrated by slow ambient drying in a glove box over a period of 12 h. “Liquid” cisplatin formulation was found to remain stable over a period of two years, without any noticeable visual residue. A schematic of the customized RESS process is shown in Figure 1.

### 2.2. Characterization of “Liquid” Cisplatin

“Liquid” cisplatin samples were extensively characterized for their microstructure and chemical composition. Ex vivo studies were carried out to determine cisplatin bio-efficacy. Optical characterizations of drop casted “liquid” cisplatin films were acquired using Nikon Eclipse LY100POL optical microscope (Nikon Instruments Inc., Melville, NY USA). Surface morphology of drop-casted and ambiently dried “liquid” cisplatin samples were analyzed by a field emission scanning electron microscope (FE-SEM, Quanta FEG 450, Thermo Fisher Scientific, Waltham, MA, USA)). The chemical composition of these samples was determined using energy dispersive X-ray spectroscopy (EDS analysis) at a 10 kV accelerating voltage and a working distance of 10 mm under high-vacuum conditions. Atomic force microscopic characterizations of these samples were performed using Agilent 5500 atomic force microscope (Keysight Technologies 5500 AFM, Keysight Technologies, Colorado Springs, CO, USA) in non-contact mode. A set point of 1.5 V was kept constant for these measurements. The topographic scans were collected with a scan speed of 0.30 Hz for 512 points/line. These scans were postprocessed by Gwyddion 2.64 software, an SPM data-visualization and -analysis tool [13]. Structural Characterization of ambiently dried “liquid” cisplatin samples were performed by X-ray diffraction (XRD) using a PANalytical Empyrean X-ray diffractometer (Malvern Panalytical, Enigma Business Park, Malvern, UK). The diffractometer was equipped with a Cu anode and the X- ray tube was operated at 45 kV and 40 mA using Bragg−Brentano geometry.

“Liquid” cisplatin samples on Si substrates were also characterized using Confocal Raman, and X-ray photoelectron spectroscopy (XR-XPS) techniques. We used a WiTec Alpha300, confocal Raman microscope (WITec Wissenschafliche Instrumente and Technologie Gmbh, Ulm, Germany) at 532 nm excitation. The compositional analysis of “liquid” cisplatin was performed by XPS (AXIS Ultra DLD, Kratos Analytical Ltd., Manchester, UK). XPS survey spectrum was used for chemical identification and High-resolution XPS (HR-XPS) spectrum for core-level binding energies. Al Kα (1486.6 eV) monochromatic source with binding energy of 1486.6 eV was used to excite the photoelectrons. XPS survey spectrum was collected at a pass energy of 160 eV and high-resolution XPS spectra of the elements (Pt 4f and Cl 2p) were collected using a pass energy of 20 eV with a step size of 0.1 eV. HR XPS studies to confirm the binding energy location and oxidation states. Carbon 1s peak at 284.6 eV with ±0.1 eV accuracy was used as the reference spectrum for these studies. The base pressure of the main chamber was maintained at 10^–8^ bar during these XPS measurements. HR-XPS data were post-processed with Gaussian curve fitting procedure [22].

Platinum quantification analysis was also conducted using an inductively coupled plasma mass spectrometry (7800 ICP-MS, Agilent Technologies, Santa Clara, CA, USA) equipped with the standard High Matrix Introduction (HMI) system and an optional Agilent Integrated Sample Introduction System (ISIS 3). Sample handling was automated using the Agilent SPS 4 autosampler. The ICP-MS setup featured a standard sample introduction system, which included a micro-mist concentric nebulizer, a quartz spray chamber and a quartz torch with a 2.5 mm inner diameter injector. The cones utilized were made of nickel, with a Cu-core sampler plated with nickel. Additionally, the Agilent argon gas humidifier was employed to the carrier (nebulizer) gas to prevent salt accumulation on the nebulizer. Standard tuning solutions were introduced at a 1:1 ratio through 0.76 mm inner diameter tubing for both the internal standardization (ISTD) and carrier/sample streams. These parameters were automatically adjusted based on the expected composition of the samples under investigation. The five target analytes (nickel, copper, zinc, cadmium and lead) were measured in helium (He) collision mode. This uncomplicated single-tune step method utilizing He mode consistently reduced or eliminated common polyatomic interferences by employing kinetic energy discrimination (KED). The ICP-MS operated at a forward power of 1600 Watts, with an auxiliary gas flow rate of 0.7 L min^−1^, nebulizer gas flow rate of 0.8 L min^−1^ and 10 channels per atomic mass unit (amu), with an integration time of 0.5 ms, respectively. The ICP-MS was set to detect Pt 195 signal. Initially, standard cisplatin concentrations were prepared in deionized water (DI water) and the standard calibration plot was generated. Finally, the quantification of “liquid” cisplatin samples was performed using Pt standards procured from Sigma Aldrich. The system was configured with I Mass Hunter 4.5 workstation software for 7800 ICP-MS (Version C.01.05 Build 588.3 MM_3_ software), which enabled rapid system setup for a specific method, auto-optimization, tuning tools and a comprehensive system for status monitoring to ensure consistent and high-quality results. Additionally, it facilitated the development of new methods tailored to the sample type and their intended applications.

### 2.3. Liquid In Situ TEM Imaging of “Liquid” Cisplatin

Talos F200X G2 TEM (a scanning Transmission Electron Microscope, Thermo Fisher Scientific, Waltham, MA, USA) operated at an accelerating voltage of 200 kV was used to characterize “liquid” cisplatin in a specially designed liquid cell attachment. It is equipped with CETA 16M camera with a lattice-fringe resolution of 0.14 nm. Liquid TEM samples are prepared in K-kit wet cell liquid TEM sample holder chamber which is a MEMS technology-based single use sample holder used for liquid specimen with a sealable micro-channel between two silicon nitride membranes supported by silicon substrates. The inside of micro-channel was coated with silicon nitride as well. It had two silicon nitride rectangular windows of 300 µm × 25 µm with a channel height of 0.2–5.0 µm to enable imaging and analysis of “liquid” cisplatin samples. It included a copper TEM aperture grid to mount the sealed wet cell and was compatible with most standard FESEM and STEM sample holders as well as detectors. K-kit cell could also be used in dry mode for standard TEM imaging. It offered an alternate mode of imaging to enable control of various gas as well as vapors and avoid TEM chamber contamination and/or sample contamination.

K-kit wet cell liquid TEM sample holders were purchased with a set of dedicated assembly tools to facilitate careful control over the liquid sample preparation process. For example, each kit was supplied with a hollow copper TEM aperture grid and the wet cell was mounted on a microchannel loading rod. K-kit channel opener was used to open the microchannel of the liquid TEM sample holder mounted on the microchannel-loading rod and 10 µL of “liquid” cisplatin sample was placed on the sample loading stand and the opened microchannel was then dipped into the sample. The capillary forces pulled the liquid sample into the microchannel of liquid TEM sample holder, which was then unloaded from the microchannel-loading rod and placed on the K-kit gluing stand in a vertical position. Vacuum compatible epoxy resins (mounting and sealing) were used to seal the open ends of the “liquid” cisplatin sample-loaded micro-channels with K-kit needle pen. A hollow copper TEM aperture grid was fastened on the sealed wet liquid TEM cell and the wet liquid TEM samples were placed in a vacuum desiccator. For 2–3 h. The “liquid” cisplatin containing samples were then loaded in a K-kit sample holder that was mounted on a conventional TEM sample holder for imaging. High-resolution images of periodic structures were analyzed using TEM Imaging and Analysis software (TIA software—TALOS F200X).

### 2.4. In Vitro Studies

Cell culture. Human adenocarcinoma lung cancer cells (ATCC no. CCL-185) were cultured in RPMI 1640 medium supplemented with 10% fetal bovine serum (FBS; GE Healthcare Life Sciences, Chicago, IL, USA) and 1% penicillin/streptomycin (Sigma-Aldrich). The cells were maintained in a 5% CO_2_ humidied atmosphere at 37 °C. Upon reaching ∼90% confluence, the cells were harvested uing 0.25% trypsin-EDTA (Sigma-Aldrich) and subsequently used for experiments or propagated further.

Preparation of cell-treatment solutions. A 10 mM stock solution of standard cisplatin was prepared by dissolving the compound in distilled water, followed by sonication to ensure full dissolution. This stock solution was then diluted in serum-free RPMI 1640 medium to obtain final cisplatin concentrations of 10–500 µM. Similarly, stock solutions of “liquid” cisplatin (NC, 75 mM) were diluted in serum-free RPMI 1640 to obtain solutions with the same concentration range as cisplatin.

Cell viability/toxicity assays. Cell viability was assessed using the CellTiter 96 Aqueous One Solution (MTS) assay (Promega, Mannheim, Germany). This assay is based on the reduction of the MTS tetrazolium compound by viable cells to generate a soluble formazan dye. The reduction of MTS is attributed to NAD(P)H-dependent dehydrogenase enzymes in metabolically active cells [23,24,25]. A549 cells were seeded at a density of 5 × 10^3^ cells/well in 100 µL of complete RPMI 1640 media in standard 96-well plates. After culturing for 24 h at 37 °C in 5% CO_2_, the growth medium was replaced with 100 µL of serum-free medium containing either standard cisplatin or NC at the desired concentrations and cells were incubated for 48–96 h at 37 °C. Thereafter, 10 µL MTS reagent was added into each well and incubated for 2 h at 37 °C. Finally, cell viability was determined by measuring the absorbance of the formazan product at 490 nm on a Synergy H1MF Multi-Mode microplate reader (BioTek Instruments, Inc., Winooski, VT, USA). Cells treated with vehicle alone were used as a control, and wells containing only the medium served as blank. The reduction of MTS was calculated from the ratio of the absorbance of treated wells to control wells.

The fraction of cells undergoing apoptosis following treatments was determined using the Dead Cell Apoptosis assay, in which FITC-conjugated annexin V detects apoptotic cells, while propidium iodide (PI) was used as a counterstain to distinguish between apoptosis and necrosis [26,27]. The assay was carried out as previously reported [28,29,30]. Briefly, A549 cells were seeded at a density of 1 × 10^6^ cells/well in complete media in 6-well plates and incubated for 24 h at 37 °C in 5% CO_2_. The cells were then treated with 50 µM standard cisplatin or NC for an additional 72 h. Subsequently, the cells were washed with ice-cold PBS, harvested using 0.25% trypsin-EDTA, centrifuged at 1000× *g* for 5 min and then re-suspended in 1× annexin-binding buffer (10 mM HEPES, 140 mM NaCl, 2.5 mM CaCl_2_, pH7.4). To 100 µL of cell suspension, 5 µL of FITC-conjugated annexin V and 1 µL of PI (100 µg/mL) were added, followed by incubation at room temperature for 15 min. Afterwards, 400 µL of 1× annexin-binding buffer was added to the stained cells and fluorescence was measured using flow cytometry (BD FACS Aria III cell sorter, BD Biosciences, San Jose, CA, USA). A total of 10,000 cells/sample were analyzed using the green channel for FITC-conjugated annexin V and red channel for PI, and the fractions of cells undergoing early or late apoptosis were quantified using the FlowJo v 10 software.

Statistical analysis. Data are presented as the mean ± standard deviation of at least three biological replicates (i.e., *n* ≥ 3). All statistical analyses were carried out using the Prism 7.0 software (GraphPad Software, Inc., La Jolla, CA, USA). To determine statistical significance between two groups, an unpaired *t*-test was used, while among three or more groups, one-way analysis of variance (ANOVA) followed by Dunnett’s or Tukey’s *post hoc* test was used. *p* < 0.05 was considered to be statistically significant.

## 3. Results and Discussion

### 3.1. Preparation and Characterization of “Liquid” Cisplatin

**Figure 2:**
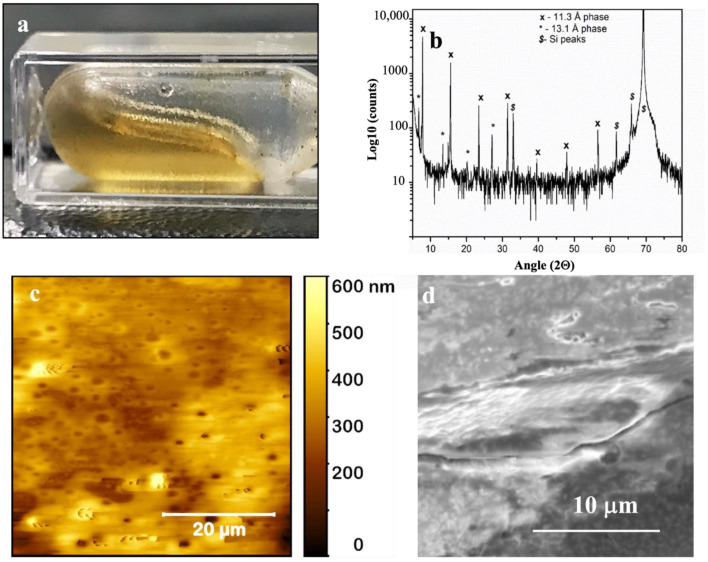
Analytical material characterization of “liquid” cisplatin. (**a**) A photograph of “liquid” cisplatin stored in a cuvette. (**b**) X-ray diffraction (XRD) spectrum collected for drop casted and dried sample of “liquid” cisplatin. (**c**) Surface topography of “liquid” cisplatin drop-casted and dried on a silicon substrate by Atomic Force Microscopy (AFM). (**d**) Scanning electron microscopy (SEM) images collected for “liquid” cisplatin drop-casted and dried on a silicon substrate.

Our experiments resulted in the collection of a viscous amber-colored liquid with honey-like consistency (Figure 2a) that is referred to as “liquid” cisplatin in this manuscript. In this section, we will demonstrate that “liquid” cisplatin comprises a stable network of highly solvated nanoclusters of cisplatin. In Figure 2b, we show the XRD for “liquid” cisplatin dried as a thin film on a silicon wafer. We observed two highly ordered superlattice structures of cisplatin nanoclusters with respective d-spacings of 11.3 Å (7 orders) and 13.1 Å (4 orders) implying an almost single crystalline quality. Since cisplatin is a coplanar van der Waals molecule, it is logical to surmise that these nanocluster arrays comprise molecular cisplatin sheets. What is not yet clear is if the nanocluster arrays were present in “liquid” cisplatin, in its liquid state prior to being dried as a film on the silicon substrate. The viscous, honey-like consistency of “liquid” cisplatin seems to indicate that they were likely present in “liquid” cisplatin itself, probably as water solvated cisplatin nanoclusters, resulting in significant intermolecular stearic hindrance, which would explain the viscous solution. It is very interesting to note that “liquid” cisplatin did not exhibit the standard XRD spectra associated with crystalline cisplatin. The characteristic diffraction peaks obtained for standard cisplatin are 14.3°, 17°, 22.3°, 37.8°, 44°, 64.3° and 77.4° as shown in Figure S1 (PDF ID 00-050-0643).

This is probably due to the very small number of cisplatin molecules in each nanocluster and we will discuss this in more detail when we examine our liquid in situ TEM characterization data later in this manuscript. Figure 2c,d show the AFM and FE-SEM images collected for “liquid” cisplatin drop-casted and dried on a silicon substrate, both confirming the absence of any detectable particles/crystals in “liquid” cisplatin (Figures S2 and S3).

**Figure 3:**
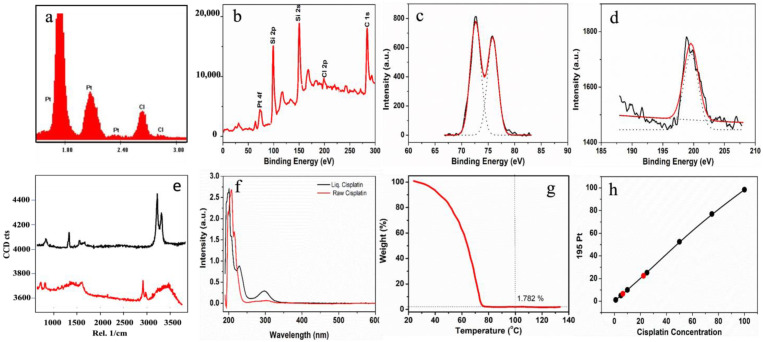
Analytical characterizations of “liquid” cisplatin. (**a**) Energy Dispersive X-Ray Spectroscopy (EDS) collected from a region of the “liquid” cisplatin film (shown in Figure 2d). (**b**) XPS survey spectrum of “liquid” cisplatin film drop-casted and dried on silicon substrate. (**c**,**d**) High-resolution X-ray photoelectron spectroscopy (HR-XPS) spectra collected in the binding energy range of Pt 4f and Cl 2p, respectively. HR-XPS were deconvoluted with Gaussian curve fitting. (**e**) Confocal Raman spectra collected for standard cisplatin powder and “liquid” cisplatin using 532 nm laser. (**f**) UV-Vis absorption spectra collected for standard cisplatin powder and “liquid” cisplatin, (**g**) Thermogravimetric analysis (TGA) of “liquid” cisplatin. (**h**) Inductively coupled plasma mass spectrometry (ICP-MS) data collected for “liquid” cisplatin.

The chemical composition of “liquid” cisplatin was determined by EDS, XPS, Confocal Raman Spectroscopy, UV-VIS absorption spectroscopy, Thermogravimetric analysis (TGA) and ICP-MS, respectively (Figure 3). EDS spectrum for “liquid” cisplatin is collected using EDS attachment of FE-SEM as shown in Figure 3a. The presence of platinum and chlorine in EDS data confirmed that the “liquid” cisplatin sample consists of Pt and Cl similar to the standard cisplatin [21]. In Figure 3b, we show the XPS survey spectrum of “liquid” cisplatin sample drop-casted on silicon substrate and dried under ambient conditions. The data which were collected for the binding energy range of 0 to 300 eV revealed several well-resolved peaks corresponding to Pt 4f, Si 2p, Si 2s, Cl 2p and C 1s at the binding energy values of 75 eV, 100 eV, 150 eV, 200 eV and 285 eV, respectively. The presence of Pt and Cl are due to cisplatin and the Si binding energy peaks are due to the silicon substrate. The exact binding energy location for Pt 4f and Cl 2p elements were determined by collecting the high-resolution XPS (HR-XPS) spectra in the range of 65–85 eV and 192–205 eV shown in Figure 3c and 3d, respectively. We observed two main peaks located around 74 eV and 77 eV, respectively. Deconvolution of HR-XPS spectrum revealed that these peaks were the two doublets of 4f_7/2_ (72.9 and 74.2) and 4f _5/2_ (76.2 and 77.5 eV) corresponding to Pt^2+^ and Pt^4+^ oxidations states, respectively. Cisplatin is normally an oxidation state II, 16 electron square planar complex. This would correspond to the Pt(II) signal. The presence of the Pt(IV) signal is likely due to environmental oxidation of the Pt(II) complex. Cisplatin from its solubilized state is perhaps more sensitive to environmental oxygen. HR-XPS data of Cl 2p showed one broad peak centered around 198 eV. The precise binding energy locations were identified by deconvolution of the experimental curve at 198.5 eV and 199.8 eV corresponding to Cl 2p^3/2^ and Cl 2p^1/2^, respectively. The presence of the broad peak of Cl 2p^3/2^ from 198.5 to 199 eV corresponds to the metal chloride formation. The binding energy difference between Cl 2p^1/^to Cl 2p^3/2^ is found to be 1.49 due to spin orbit coupling to Cl 2p. Generally, high-resolution Cl 2p spectrum peak mainly arises either from the inorganic chlorine (peaks with binding energy < 199 eV) and organic chlorine (binding energy > 200 eV). In current data, the binding energy peaks are found to be <200 eV thereby confirming that “liquid” cisplatin does not have any organic Cl peak in this spectrum [31]. Overall, these investigations confirm that the presence of Pt and Cl in “liquid” cisplatin are due to cisplatin and that it had retained the chemical and structural identity of standard cisplatin [21]. 

In Figure 3e we show the Raman spectra of “liquid” cisplatin and compare it with that of the standard cisplatin powder. The sharp bands between 3200 and 3400 cm^−1^ in the standard cisplatin crystalline powder are attributed to NH stretches. Those same bands have shifted to lower energy between 2900 and 3100 cm^−1^ for the “liquid” cisplatin. The shift of the Raman bands to lower energy and their broadening relative to those in crystalline cisplatin solid are to be expected and are consistent with the greater intermolecular interactions, including hydrogen bonding. The very broad band between 3000 and 3600 cm^−1^ is due to water and the large bandwidth is because of hydrogen bonding in the solvated species due to the water molecules. This is consistent with the observations made by Tuschel [32], who demonstrated that absence of solvation due to water molecules minimizes the molecular interactions and collisional broadening, resulting in the NH symmetric stretching mode shifting to higher energy with a narrower distribution of vibrational states. The Raman spectra unequivocally demonstrated the dominant presence of hydrogen bonding from water molecules to create a highly solvated cisplatin nanoclusters framework in “liquid” cisplatin.

In Figure 3f, we compare the UV-Vis absorption spectra of “liquid” cisplatin with the standard cisplatin powder. The broad absorption band around 300 nm for the standard cisplatin has significantly narrowed into a highly resolved band. We also observed an additional, highly resolved, strong band that was blue shifted by 60 nm at 240 nm for “liquid” cisplatin. These two sharp UV-Vis bands imply the presence of a bi-modal distribution of nanoclusters in “liquid” cisplatin that were also observed in our XRD observations of the drop-casted and dried film, namely, the 11.3 Å and 13.1 Å sized cisplatin nanoclusters. In Figure 3g, we show the TGA results for “liquid” cisplatin, showing a residual platinum concentration of 1.782% (wt. %) corresponding to a cisplatin concentration of 27.4 mg/mL which is **27 times** greater than its normal water solubility. The platinum concentration in “liquid” cisplatin was also determined using ICP-MS spectrometry (Figure 3h). Initially, 2.65 µL and 9.52 µL of “liquid” cisplatin were diluted into 10 mL of water to obtain a analyte for ICP-MS and used to quantify the platinum content of the “liquid” cisplatin in solution. The ICP-MS analysis revealed measured concentrations of Pt 195 signal at 6.46 and 22.36 ppm, corresponding to sample “liquid” cisplatin concentrations of 19.9 µM and 71.4 µM, respectively in the calibration plot (Figure 3h). Both sets of results confirm that the original concentration of the “liquid” cisplatin was 22.6 mg/mL which is slightly lower than that measured by TGA.

The chemical structure of “liquid” cisplatin was also investigated by using the Fourier-transform infrared (FTIR) spectroscopy. FTIR spectroscopic data collected for “liquid” cisplatin are listed in Figure S4. FTIR spectrum of standard cisplatin powder showed the characteristic amine stretching peak (3279 cm^−1^ and 3203 cm^−1^), the asymmetric amine bending (around 1640 cm^−1^, 1535 cm^−1^) and symmetric amine bending (1293 cm^−1^) [33,34,35]. “liquid” cisplatin showed a red shift in the symmetric amine stretching peak to 3341 cm^−1^. In asymmetric amine bending mode “liquid” cisplatin revealed only one band at 1640 cm^−1^ while other two bands located at lower wavenumber at 1535 cm^−1^ and 1293 cm^−1^ are absent [36,37]. The reasons for the observed red shift in the characteristic amine stretching and absence of asymmetric amine bending in “liquid” cisplatin are currently under investigation. Differential Scanning Calorimetry (DSC) data are reported in Figure S5.

### 3.2. Liquid TEM Analysis of “Liquid” Cisplatin

**Figure 4:**
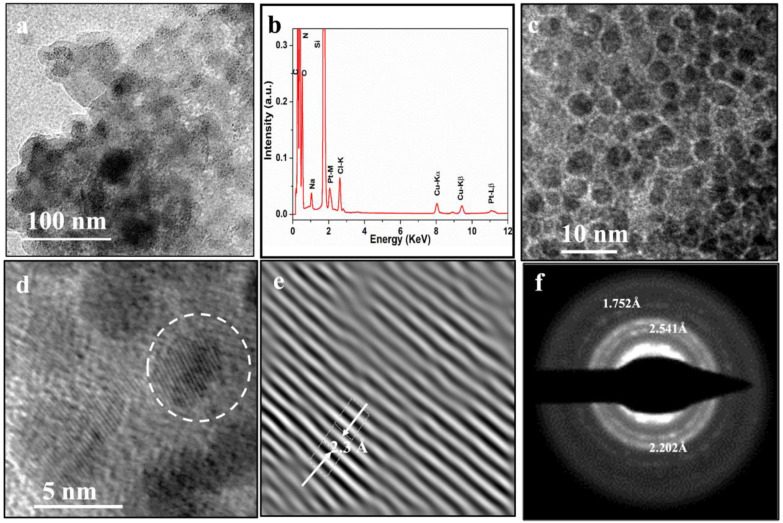
Liquid cell in situ TEM demonstration of nanostructured “liquid” cisplatin. (**a**) Low-magnification image of a “liquid” cisplatin region. (**b**) Energy dispersive X-ray spectroscopy (EDS) data collected from a region of “liquid” cisplatin in Figure 4a confirming cisplatin chemical identity. (**c**) High-resolution liquid TEM image of “liquid” cisplatin from the white circle region provides a compelling proof of the presence of self-assembled and ordered cisplatin nanocluster structures. (**d**) Higher-magnification image of a region from (**c**), showing lattice image of metallic platinum lattice in a cisplatin nanocluster. (**e**) Inverse Fast Fourier Transform (IFFT) image of the region in the white circle in the Figure 3d showing a lattice spacing of 2.3 Å corresponding to metallic platinum (*d* _Pt(111)_ = 2.3 Å) in the “liquid” cisplatin. Figure (**f**) is the selective area electron diffraction pattern collected of “liquid” cisplatin confirming the platinum lattice.

Intrigued by our Raman observations of “liquid” cisplatin, we decided to search for direct evidence for a nanoclusters framework in “liquid” cisplatin using the so-called “liquid TEM” characterization techniques. In Figure 4, we report the transmission electron microscopy characterization results of “liquid” cisplatin enclosed in a K-kit wet cell liquid TEM sample holder chamber. In bright field mode of imaging, the incident beam direction is normal to the specimen surface and the atomic positions were realized by high-resolution TEM imaging in the same regions. The detailed procedure of TEM liquid cell sample preparation is described in the experimental section. Figure 4a is a low-magnification image of a “liquid” cisplatin region. Figure 4b shows energy dispersive X-ray spectroscopy (EDS) data collected from a region of the “liquid” cisplatin in Figure 4a. The presence of Pt and Cl binding energy peaks in EDS data once again confirmed that it is cisplatin. High-resolution liquid TEM image of “liquid” cisplatin (Figure 4c) provided unequivocal proof for the presence of cisplatin nanoclusters framework in the liquid phase itself. Figure 4d is a higher-magnification image of a region selected from Figure 4c, showing lattice image of crystalline platinum lattice in a cisplatin nanocluster. In Figure 4e, we show the inverse Fast Fourier Transform (IFFT) constructed from the marked region shown in Figure 4d revealing a highly ordered structure with a lattice spacing of 2.3 Å as indicated by the white dashed lines. The measured lattice spacing of 2.3 Å is due to metallic platinum (*d* _Pt(111)_ = 2.3 Å) in the “liquid” cisplatin. Figure 4f is the selective area electron diffraction (SAED) pattern of “liquid” cisplatin shown in Figure 4a confirming the platinum lattice planes.

In summary, we have demonstrated the following: the chemical identity of “liquid” cisplatin to be cisplatin, primarily comprising a highly solvated bi-modal cisplatin nanoclusters framework resulting in a viscous (aqueous) liquid form that is stable for over a year at room temperature. The amount of solubilized cisplatin in water was **27 times** greater than of standard cisplatin.

### 3.3. Cell Viability and Apoptosis Studies

**Figure 5:**
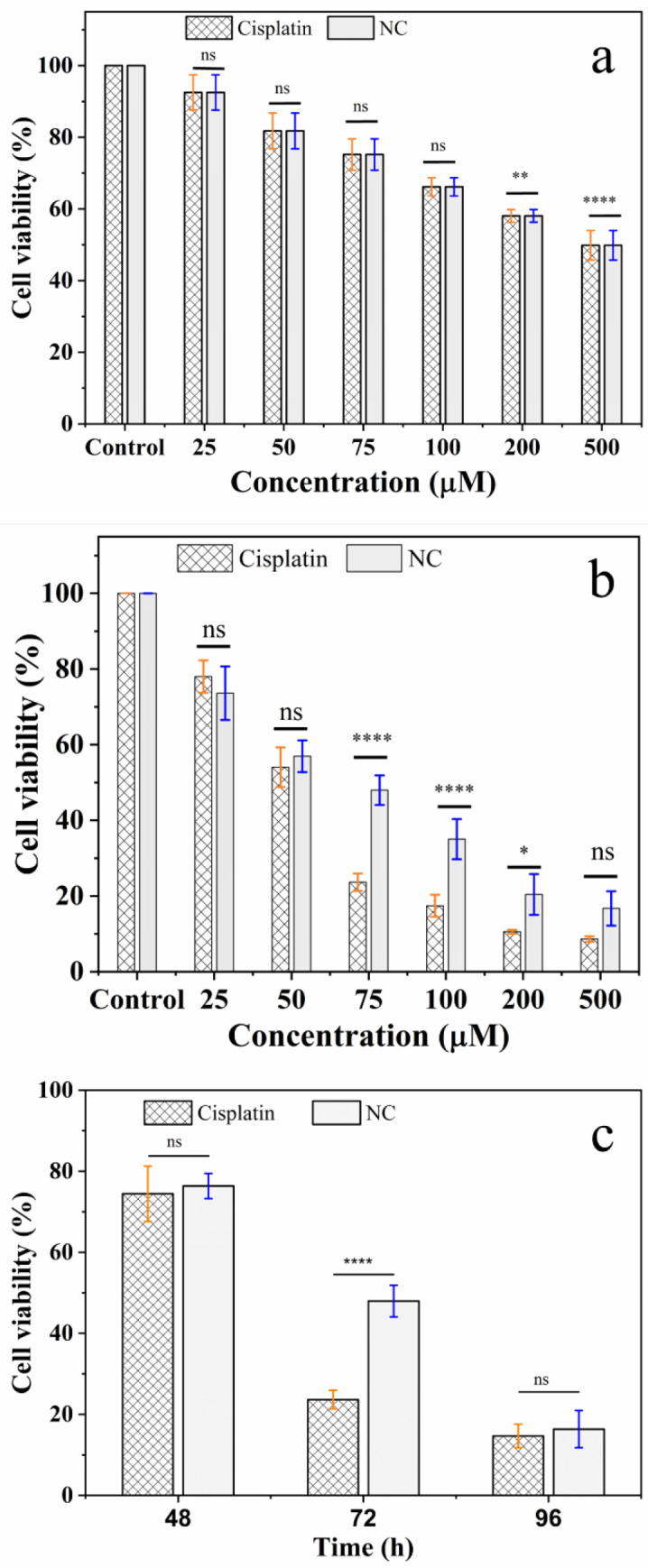
Cytotoxic effects of standard cisplatin and “liquid” cisplatin. (**a**,**b**) Dose dependence of toxicity of cisplatin and “liquid” cisplatin to human lung adenocarcinoma A549 cells. The cells were treated with increasing concentrations of standard cisplatin (Cisplatin) and “liquid” cisplatin (NC) for 48 h (**a**) or 72 h (**b**), and viability was measured using the MTS assay. (**c**) Time dependence of cisplatin and “liquid” cisplatin cytotoxicity. Based on the IC_50_ (75 μM) calculated for NC, toxicity of this concentration to A549 cells were compared at different incubation times (48–96 h). Cell viability was measured using the MTS assay, with the % viability determined form the ratio of the absorbance of the treated cells to the control cells. The errors bars represent the S.D. of five independent triplet-well trials. * p < 0.05, ** *p* < 0.01, **** *p* < 0.0001 or non-significant (ns, *p* > 0.05) for comparisons among the treatment groups.

Human lung adenocarcinoma A549 cells were treated with increasing concentrations of standard cisplatin or “liquid” cisplatin, and cytotoxicity was evaluated using the MTS assay (Figure 5). As expected, standard cisplatin reduced A549 cell viability in a dose-dependent manner. At the lowest concentration of cisplatin tested (25 μM), viability of A549 cells decreased to 78 ± 4% of vehicle-treated controls, while at 75 μM cisplatin, viability decreased further to 24 ± 2% of controls, at 72 h incubation (Figure 5b). Of note, the IC_50_ value is highly dependent on a number of experimental conditions, including cell type and seeding density, as well as the choice of cell viability/toxicity assay. Nevertheless, the IC_50_ value calculated for standard cisplatin here was 30 ± 7 μM, which falls within the range of values reported in the literature [38,39,40]. Likewise, “liquid” cisplatin exhibited dose-dependent cytotoxicity. At 25 and 75 μM concentrations of “liquid” cisplatin, the cell viabilities were 73 ± 7 and 47 ± 4%, respectively, of controls at 72 h incubation (Figure 5b). The IC_50_ for “liquid” cisplatin was determined to be 75 ± 5 μM, which was higher than that of standard cisplatin. However, comparison of the cytotoxicity of the two preparations as a function incubation time (24–96 h) showed that, while “liquid” cisplatin is less potent than standard cisplatin at 72 h, at the longer incubation time of 96 h the toxicity of the two preparations was comparable (15 ± 3 and 16 ± 4% cell viability for standard cisplatin and “liquid” cisplatin, respectively) (Figure 5c). The original experiment shown in Figure 5c was again repeated after two years (Figure S6). The reproducibility of the data confirms the stability of the compound over this period.

**Figure 6:**
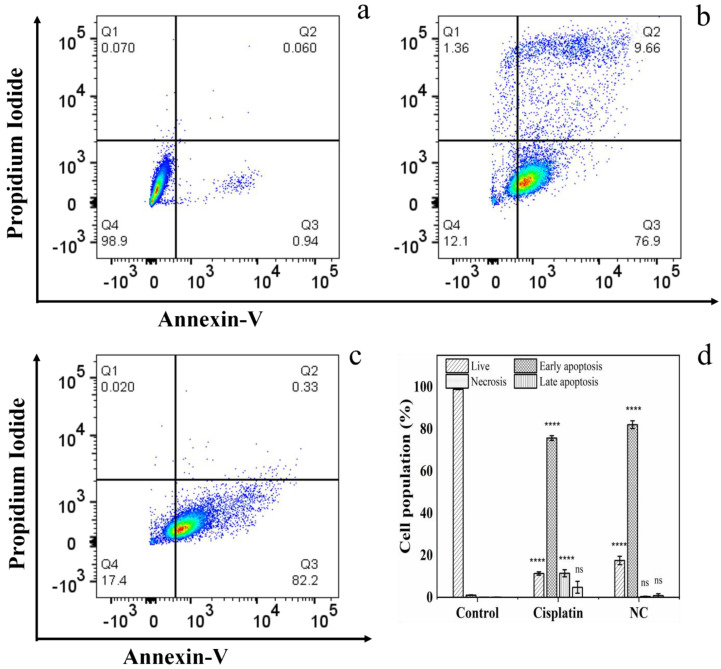
Detection of apoptotic cells following treatment with standard cisplatin and RESS-processed “liquid” cisplatin. (**a**–**c**) Flow cytometry analysis of FITC-conjugated annexin V/PI-stained A549 cells that were either untreated (control, Ctrl) (**a**), or treated with 75 μM cisplatin (standard cisplatin) (**b**) or “liquid” cisplatin (NC) (**c**) for 72 h. The four quadrants are defined as follows: annexin V^−^/PI^−^ (**bottom left**), live cells; annexin V^+^/PI^−^ (**bottom right**), early apoptotic cells; annexin V^+^/PI^+^ (**top right**), late apoptotic cells; and annexin V^−^/PI^+^ (**top left**), necrotic cells. (**d**) A summary of the incidence of early/late apoptosis and necrosis in the A549 cells treated with cisplatin and “liquid” cisplatin determined from the flow cytometry analysis of annexin V/PI staining (n = 3). The errors bars represent the S.D. of three independent trails. **** *p* < 0.0001 or non-significant (ns, *p* > 0.05) for comparisons with controls and among the treatment groups.

The cytotoxic effects of standard cisplatin and “liquid” cisplatin were further probed using FITC-conjugated annexin V and propidium iodide (PI) staining. Annexin V binds to exposed phosphatidylserines in apoptotic cells, while the membrane-impermeant red-fluorescent PI, a nucleic acid-intercalating dye, simultaneously distinguishes between apoptosis and necrosis by assessing plasma membrane integrity [26,27]. A549 cells were exposed to 75 μM standard cisplatin and “liquid” cisplatin for 72 h, and the fractions of live (annexin V^−^/PI^−^), early apoptotic (annexin V^+^/PI^−^), late apoptotic (annexin V^+^/PI^+^) and necrotic (annexin V^−^/PI^+^) cells were quantified (Figure 6). Treatment with standard cisplatin led to 76 ± 1 and 11 ± 2% of the cells undergoing early and late apoptosis, respectively. On the other hand, treatment with “liquid” cisplatin yielded a significantly higher proportion of early apoptotic cells (82 ± 2%), but a negligible fraction of cells undergoing late apoptosis. Thus, the annexin V/PI staining confirms the more gradual cytotoxic effects of “liquid” cisplatin compared to standard cisplatin observed with the MTS cell viability assay. Taken together, the cell viability/toxicity results demonstrate that “liquid” cisplatin yields a more sustained anticancer effect compared to standard cisplatin, which underscores the potential of “liquid” cisplatin as a promising candidate for further investigation and development in cancer treatment.

## 4. Conclusions

We report our discovery of a new viscous liquid form of cisplatin, an important and commonly used anticancer drug, comprising a framework of highly solvated cisplatin nanoclusters in water. “liquid” cisplatin demonstrated a **27×** greater water solubility than standard cisplatin and was stable at ambient conditions for over a year. Extensive analytical characterization confirmed that the new formulation retained the original chemical identity of cisplatin. Cell viability and apoptosis studies provided compelling evidence that “liquid” cisplatin demonstrated a more sustained anticancer effect compared to standard cisplatin. These observations underline the exciting potential of “liquid” cisplatin in development of non-invasive and highly effective novel cancer nanoplatforms.

## Data Availability

All the supporting information is available free of charge.

## Supplementary Materials

The following supporting information can be downloaded at: https://www.mdpi.com/article/10.3390/pharmaceutics16111471/s1, Figure S1: XRD characterizations of liquid cisplatin; Figure S2: AFM imaging of liquid cisplatin; Figure S3: SEM imaging of liquid cisplatin; Figure S4: FTIR spectroscopic data collected for standard cisplatin and liquid cisplatin; Figure S5: DSC of nanostructured cisplatin; Figure S6: Cytotoxic effects of standard cisplatin and “liquid” cisplatin two years after the first study reported in Figure 5c.
