# Supplementary material for: Synthesis of Self-Assembled Nanostructured Cisplatin Using the RESS Process
Source: Pharmaceutics. 2024 Nov 18;16(11):1471. doi: 10.3390/pharmaceutics16111471 (PMC11597759; doi:10.3390/pharmaceutics16111471)
Supplement: Supplementary file 1 [file pharmaceutics-16-01471-s001.zip › pharmaceutics-3298837-supplementary.pdf]

Synthesis of Self-Assembled Nanostructured Cisplatin using the RESS Process

Sudhir Kumar Sharma[†], Loganathan Palanikumar[‡], Renu Pasricha[§], Thirumurugan Prakasam[+], Mazin Magzoub[‡] and Ramesh Jagannathan[†]

[†]Engineering Division, New York University Abu Dhabi, UAE

[‡] Program in Biology, New York University Abu Dhabi, UAE

[§] Retired from Core Technology Platform, New York University Abu Dhabi, UAE

[+]Science Division, New York University Abu Dhabi, Abu Dhabi, UAE

Electronic supplementary Information

Table of Contents

Figure S1: XRD characterizations of liquid cisplatin
Figure S2: AFM imaging of liquid cisplatin
Figure S3: SEM imaging of liquid cisplatin
Figure S4: FTIR spectroscopic data collected for standard cisplatin and liquid cisplatin
Figure S5: DSC of nanostructured cisplatin
Figure S6: Cytotoxic effects of standard cisplatin and "liquid" cisplatin two years after the first study reported in Figure 5c.

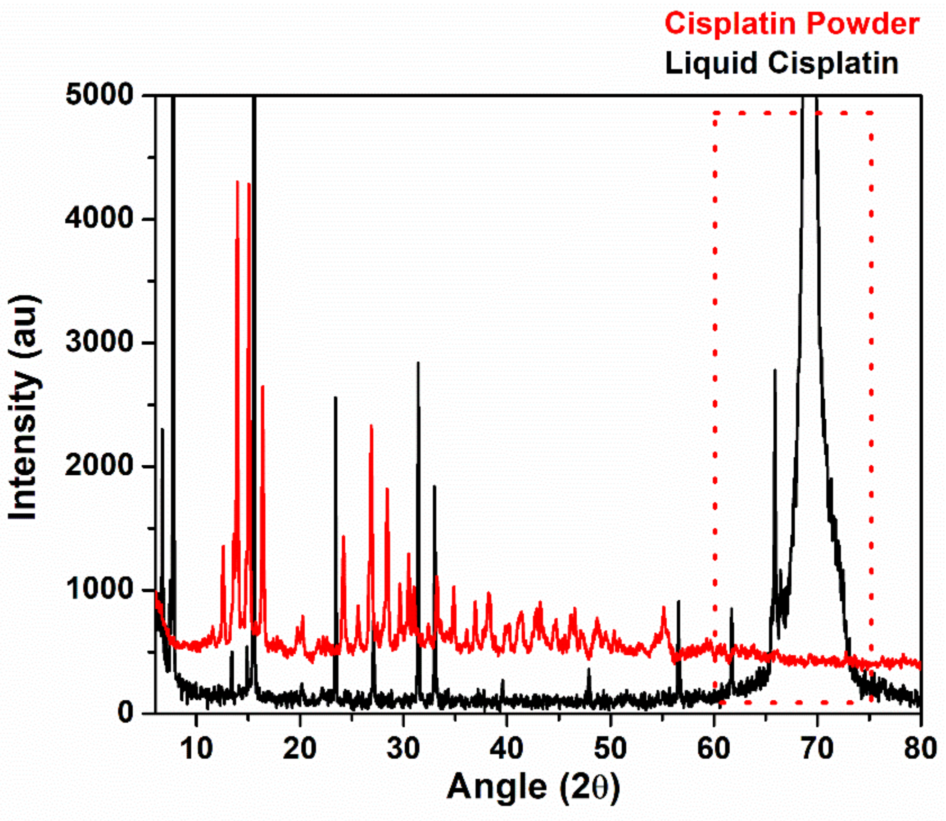

Figure S1- XRD characterization- XRD data collected for liquid cisplatin and standard cisplatin. Standard cisplatin displays the characteristic peaks of cisplatin while liquid cisplatin displays highly ordered super lattice structures.

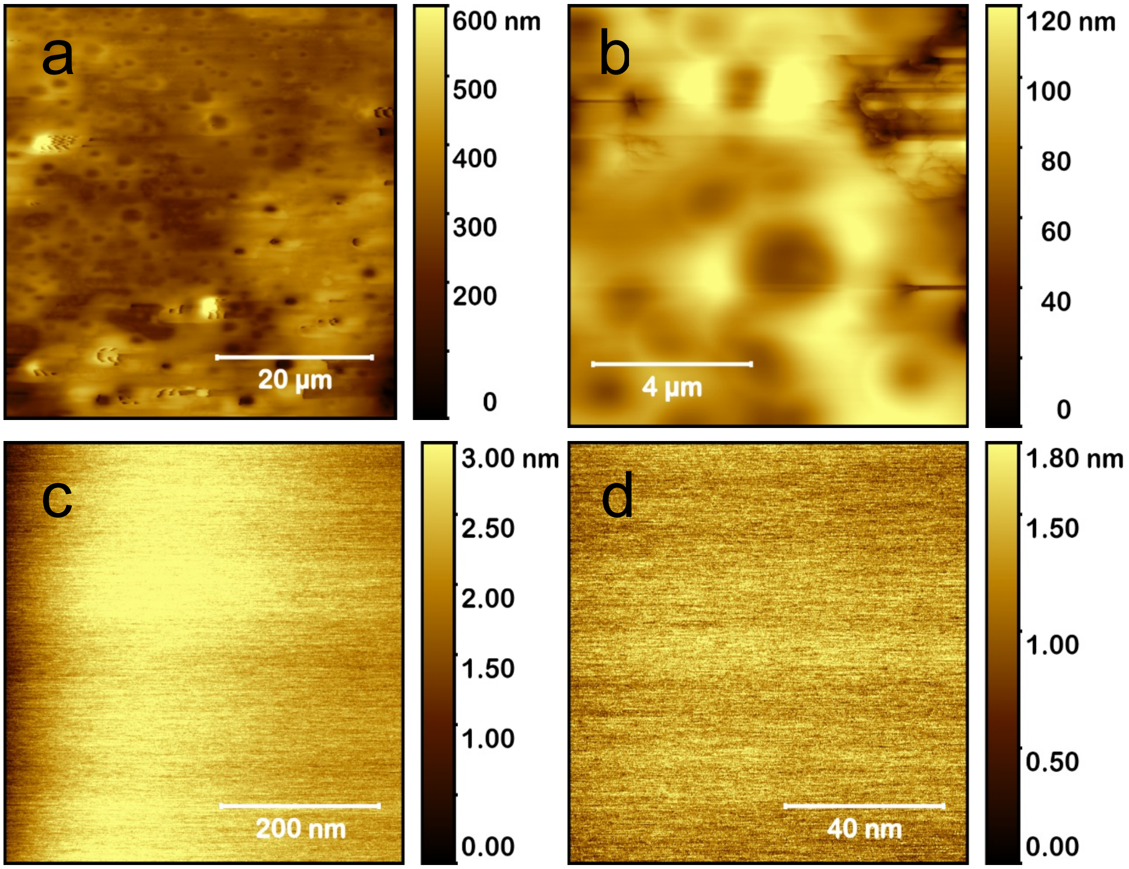

Figure S2- AFM imaging- AFM imaging performed for liquid cisplatin at a scan size of (a) 50 µm, (b) 10 µm, (c) 500 nm and (d)100 nm scan size, respectively.

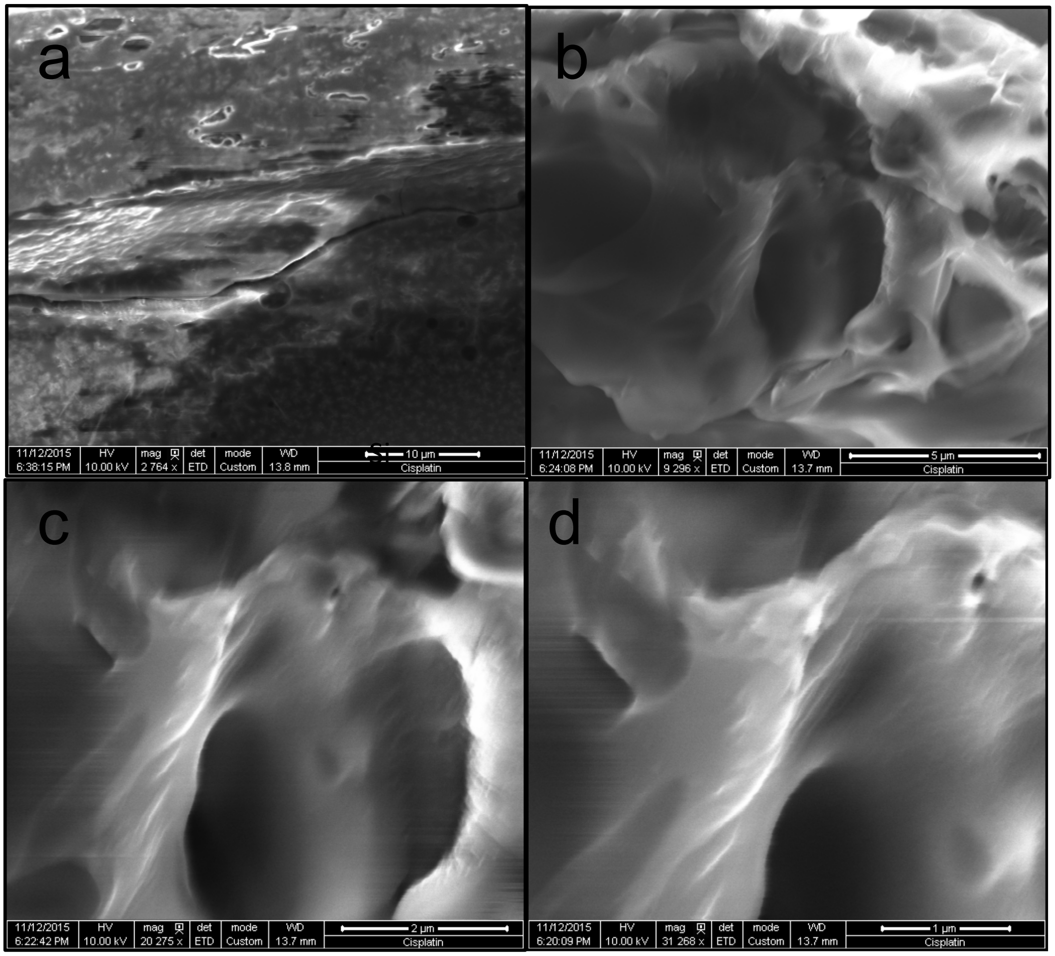

Figure S3- SEM Imaging- SEM imaging performed for liquid cisplatin at different magnifications.

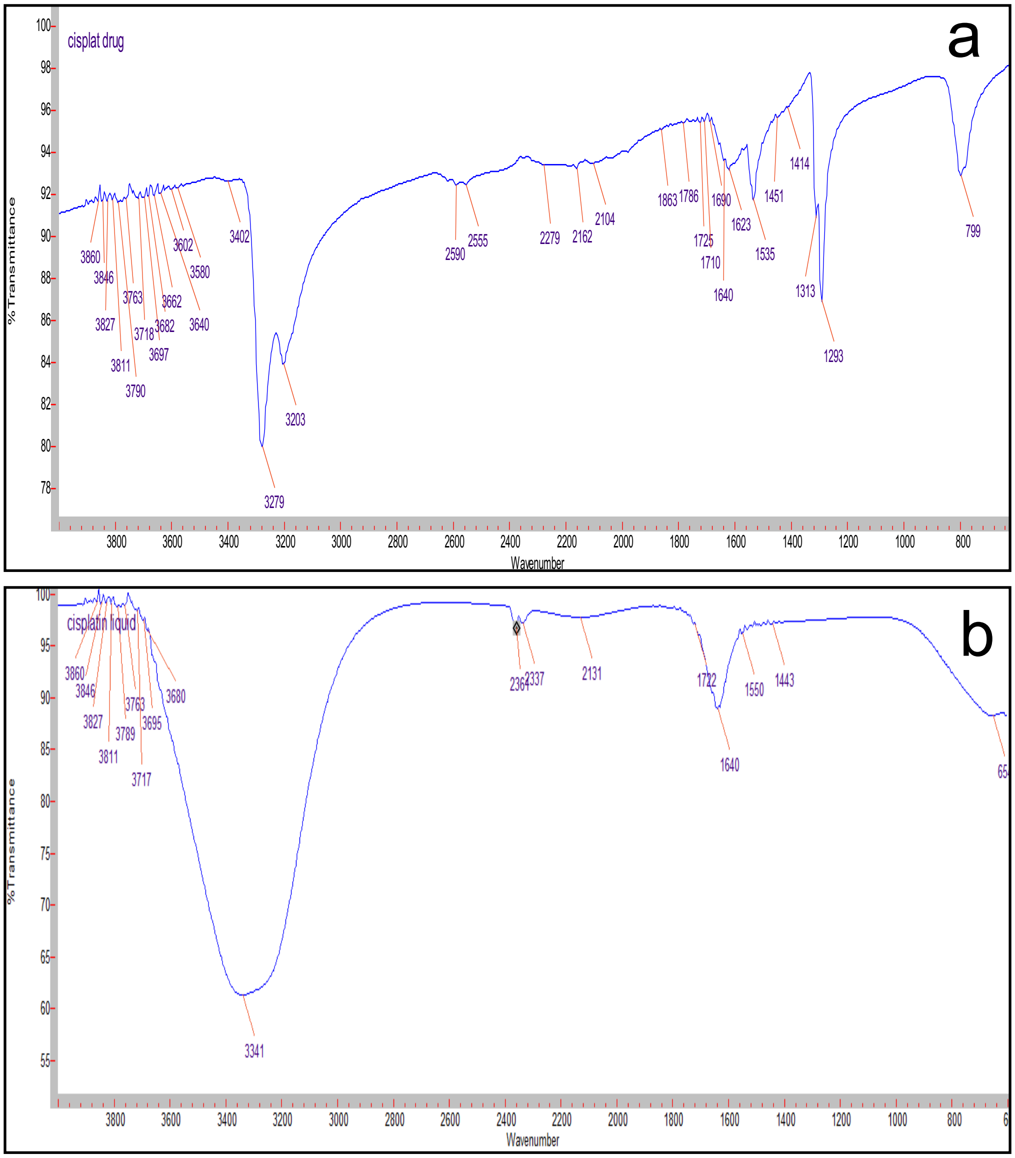

Figure S4- FTIR spectroscopic characterization- FTIR spectroscopic data collected for standard cisplatin powder and liquid cisplatin. For liquid cisplatin, we did not observe the lower energy bands located at 1535 cm^{-1} and 1293 cm^{-1}, while a red shift in the asymmetric amine band from 3279 cm^{-1} to at 3341 cm^{-1} was observed.

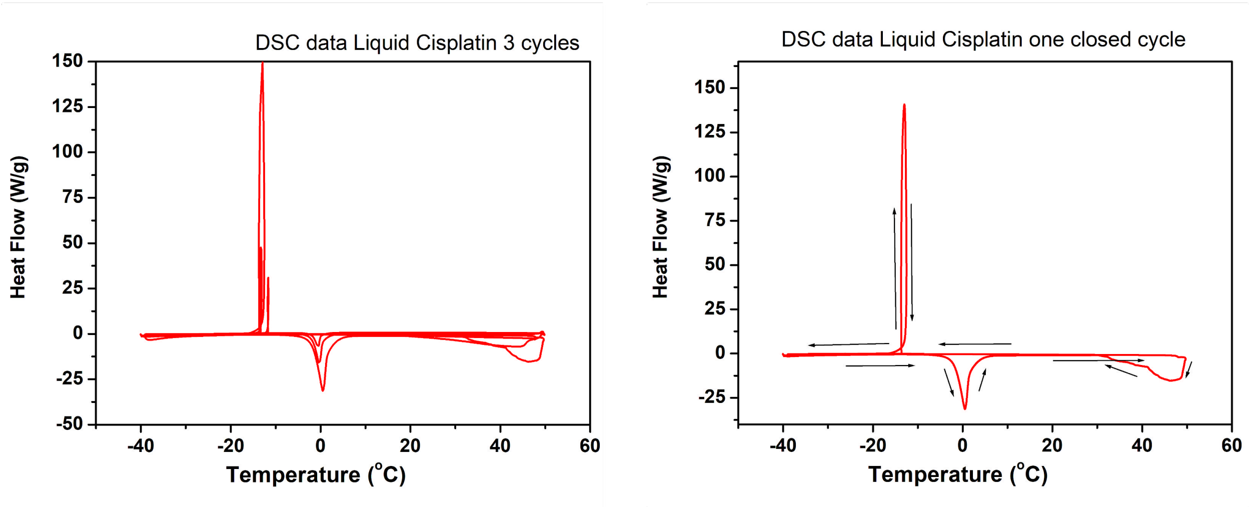

Figure S5: DSC Q200 were used to collect the closed cycle DSC data for liquid Cisplatin . 3mg drop of Liquid Cisplatin were used for these measurements with Nitrogen purging of 50 ml/min. The sample were ramped with 10°C/min from -40°C to 50°C. The sample was stabilized for 5 min at the lowest and height temperature to obtain isothermal conditions. The temperature range was selected so that the detector of DSC equipment would remain safe during measurement from liquid sample.

DSC data collected for Liquid Cisplatin sample ramped for three scans from -40°C to 50° C in heating and cooling cycles (shown in Figure a), respectively. All these scan were found to be repeatable and a highlighted version of single scan is shown in Figure b. While heating from -40°C, we observed a melting of solid water from liquid cisplatin sample around 0.3 ^{o}C and crystallization of liquid cisplatin at -13°C in the cooling cycle. In these scans, we did not notice any evaporation as DSC of liquid samples as the higher temperature range was limited by detector for liquid samples. Usually for standard Cisplatin powder sample the evaporation of water vapors are reported in literature around 100° C. [ref. Banella et al 2024 7 100436 (1-11).]. We did not notice any glass transition in liquid cisplatin samples.

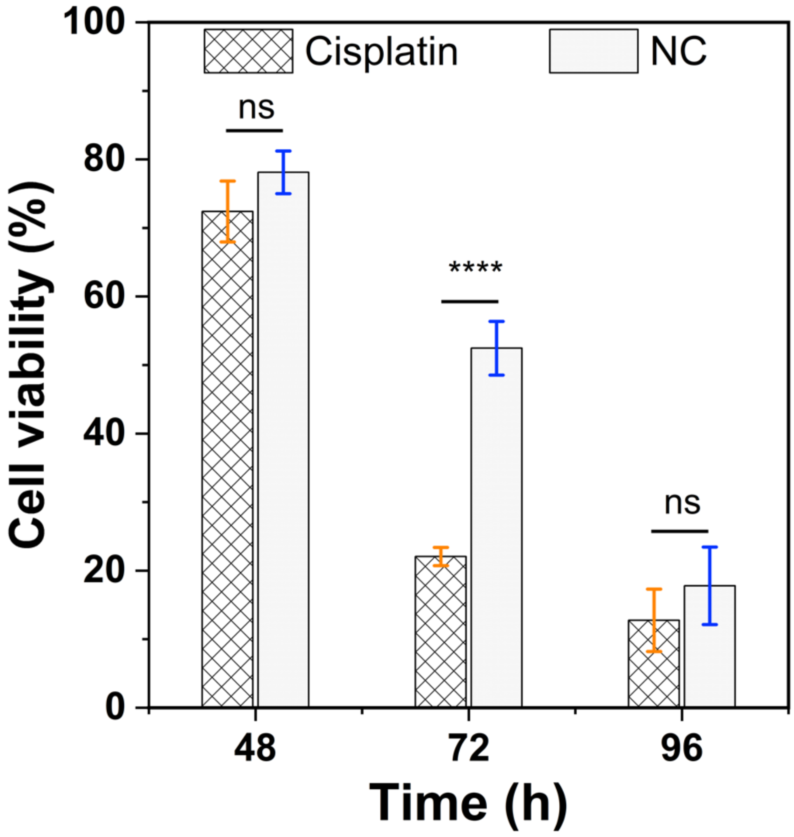

Figure S6: Cytotoxic effects of standard cisplatin and "liquid" cisplatin two years after the first study reported in Figure 5c. Time-dependence of cisplatin and "liquid" cisplatin cytotoxicity. Based on the IC_{50} (75 μM) calculated for NC, toxicity of this concentration to A549 cells were compared at different incubation times (48–96 h). Cell viability was measured using the MTS assay, with the % viability determined form the ratio of the absorbance of the treated cells to the control cells. The errors bars represent the S.D. of five independent triplet-well trials. $*P < 0.05$, $**P < 0.01$, $****P < 0.0001$ or non-significant (ns, $P > 0.05$) for comparisons among the treatment groups.
